# Structure, Properties, and Function of Glycosomes in *Trypanosoma cruzi*

**DOI:** 10.3389/fcimb.2020.00025

**Published:** 2020-01-31

**Authors:** Wilfredo Quiñones, Héctor Acosta, Camila Silva Gonçalves, Maria Cristina M. Motta, Melisa Gualdrón-López, Paul A. M. Michels

**Affiliations:** ^1^Laboratorio de Enzimología de Parásitos, Facultad de Ciencias, Universidad de Los Andes, Mérida, Venezuela; ^2^Laboratório de Ultraestrutura Celular Hertha Meyer, Centro de Ciências da Saúde, Instituto de Biofísica Carlos Chagas Filho, Universidade Federal Do Rio de Janeiro, Rio de Janeiro, Brazil; ^3^Instituto Salud Global, Hospital Clinic-Universitat de Barcelona, and Institute for Health Sciences Trias i Pujol, Barcelona, Spain; ^4^Centre for Immunity, Infection and Evolution and Centre for Translational and Chemical Biology, The University of Edinburgh, Edinburgh, United Kingdom

**Keywords:** trypanosomes, glycosomes, peroxisomes, glycolysis, metabolic networks, metabolite transport, biogenesis, drug discovery

## Abstract

Glycosomes are peroxisome-related organelles that have been identified in kinetoplastids and diplonemids. The hallmark of glycosomes is their harboring of the majority of the glycolytic enzymes. Our biochemical studies and proteome analysis of *Trypanosoma cruzi* glycosomes have located, in addition to enzymes of the glycolytic pathway, enzymes of several other metabolic processes in the organelles. These analyses revealed many aspects in common with glycosomes from other trypanosomatids as well as features that seem specific for *T. cruzi*. Their enzyme content indicates that *T. cruzi* glycosomes are multifunctional organelles, involved in both several catabolic processes such as glycolysis and anabolic ones. Specifically discussed in this minireview are the cross-talk between glycosomal metabolism and metabolic processes occurring in other cell compartments, and the importance of metabolite translocation systems in the glycosomal membrane to enable the coordination between the spatially separated processes. Possible mechanisms for metabolite translocation across the membrane are suggested by proteins identified in the organelle's membrane—homologs of the ABC and MCF transporter families—and the presence of channels as inferred previously from the detection of channel-forming proteins in glycosomal membrane preparations from the related parasite *T. brucei*. Together, these data provide insight in the way in which different parts of *T. cruzi* metabolism, although uniquely distributed over different compartments, are integrated and regulated. Moreover, this information reveals opportunities for the development of drugs against Chagas disease caused by these parasites and for which currently no adequate treatment is available.

## Introduction

**Figure 1:**
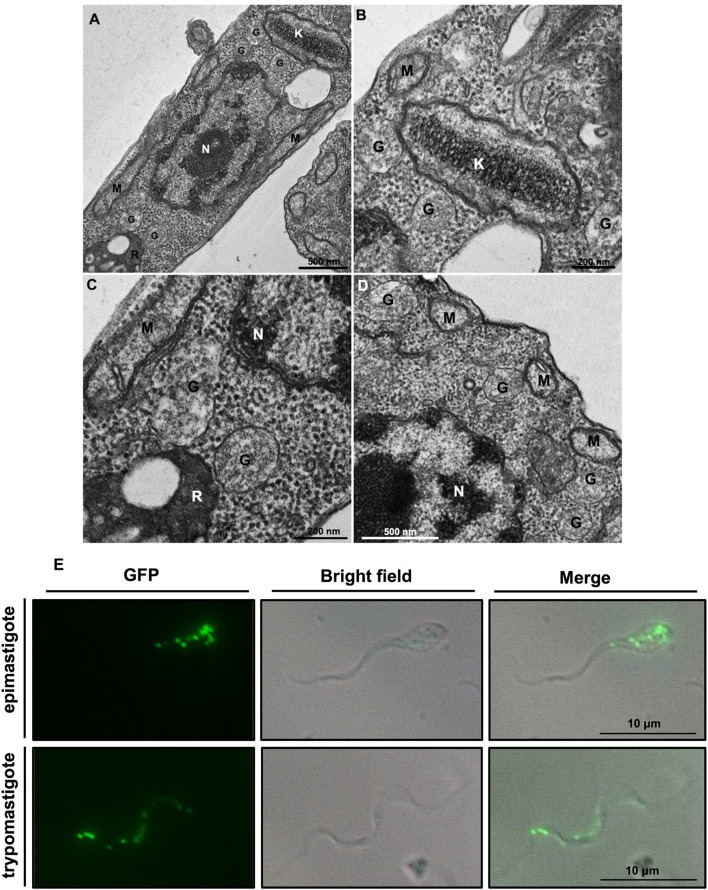
**(A–D)** Ultrastructure of *Trypanosoma cruzi* epimastigote form showing glycosomes (G) and their proximity to other cell structures, especially the kinetoplast (K) and mitochondrial branches (M). Glycosomes are also seen close to reservosomes (R); N, nucleus. **(B,C)** Are insets that show areas of **(A)** with higher magnification. Cells were processed to transmission electron microscopy as follows. Protists were fixed for 1 h in 2.5% type II glutaraldehyde (Sigma, Missouri, USA) diluted in 0.1 M cacodylate buffer (pH 7.2). Then, they were washed twice in cacodylate buffer and post-fixed (1% osmium tetroxide, 0.8% potassium ferrocyanide, 5 mM calcium chloride diluted in 0.1 M cacodylate buffer) for 1 h. After fixation, samples were washed in cacodylate buffer, dehydrated in a graded series of acetone solutions (50%, 70%, 90%, and two exchanges of 100% acetone) for 10 min each step, and embedded in Polybed resin. Ultrathin sections were stained with 5% uranyl acetate for 45 min and lead citrate for 5 min before observation in a Jeol 1200ex operating at 80 kV. **(E)** *T. cruzi* contains multiple glycosomes distributed throughout the cell body as demonstrated by fluorescent puncta in transgenic epimastigotes and metacyclic trypomastigotes expressing green fluorescent protein (GFP) containing a C-terminal PTS1 (-SKL) to target it to glycosomes.

Like other kinetoplastids, *Trypanosoma cruzi* contains peroxisome-related organelles called glycosomes (Figures 1A–D). Peroxisomes constitute a family of organelles that are present in all superphyla of eukaryotes. Despite their diversity in protein content and size, these organelles are homologous and share important features of their biogenesis and morphology and some functions (1). Glycosomes are authentic members of this family, but characterized by containing enzymes of the glycolytic and gluconeogenic pathways. In some cases, such as *Trypanosoma brucei* living in the mammalian bloodstream, glycolytic enzymes may even comprise over 90% of the glycosomal protein content. Probably, the common ancestor of the Kinetoplastea and Diplonemida sequestered these enzymes in their peroxisomes (Gualdrón-López et al., 2012a; Gabaldón et al., 2016; Morales et al., 2016).

Trypanosomatids possess multiple small glycosomes. Approximately 60–65 of these organelles with an average diameter of 0.27 μm have been reported for bloodstream-form *T. brucei*, distributed throughout the cell body, often in clusters, with the number increasing to ~120 during parasite growth up to cell division (Opperdoes et al., 1984; Tetley and Vickerman, 1991; Hughes et al., 2017), whereas 50 glycosomes have been found in different life-cycle stages of *T. cruzi* (Soares and de Souza, 1988; Soares et al., 1989) (Figure 1E). Importantly, many of the glycosomal enzymes, as well as their sequestering inside the organelles have been shown to be essential for the viability of different trypanosomatids, rendering the organelles promising targets for new drugs to be developed (Galland and Michels, 2010; Barros-Alvarez et al., 2014; Dawidowski et al., 2017). In this minireview, we will highlight some recent findings about glycosomes, particularly from *T. cruzi*. More detailed information about glycosomes can be found elsewhere (Gualdrón-López et al., 2012a; Barros-Alvarez et al., 2014; Allmann and Bringaud, 2017).

## Proteome of *T. cruzi* GLYCOSOMES

**Figure 2:**
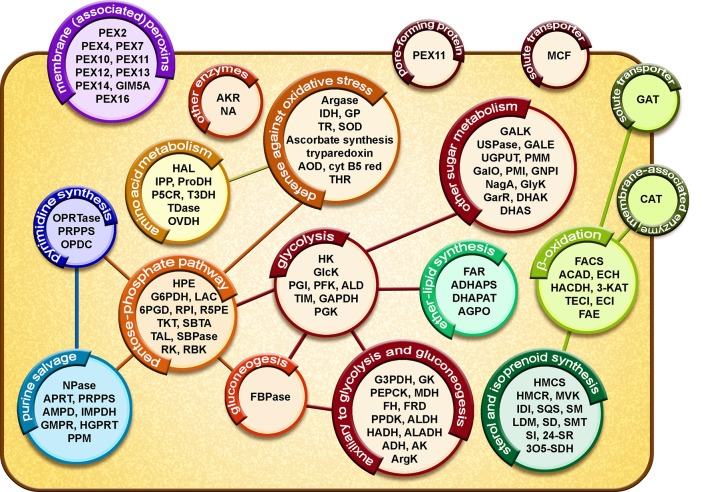
Diagrammatic representation of the glycosomal proteome of *T. cruzi* epimastigotes. Proteins involved in metabolic pathways, solute transport and glycosome biogenesis are represented in circles. Lines represent connections between different processes by exchange of metabolites. **Glycolysis**: HXK, hexokinase; GlcK, glucokinase; PGI, phosphoglucose isomerase; PFK, phosphofructokinase; ALD, aldolase; TIM, triosephosphate isomerase; GAPDH, glyceraldehyde-3-phosphate dehydrogenase; PGK, phosphoglycerate kinase. **Auxiliary to glycolysis and gluconeogenesis**: G3PDH, glycerol-3-phosphate dehydrogenase; GK, glycerol kinase; PEPCK, phosphoenolpyruvate carboxy kinase; MDH, malate dehydrogenase; FH, fumarate hydratase; FRD, NADH-fumarate reductase; PPDK, pyruvate phosphate dikinase; ALDH, aldehyde dehydrogenase; HADH, D-isomer specific 2-hydroxyacid dehydrogenase-protein; ALADH, alanine dehydrogenase; ADH, alcohol dehydrogenase; AK, adenylate kinase; ArgK, arginine kinase. **Other sugar metabolism**: GALK, galactokinase; USPase, UDP-sugar pyrophosphorylase; GALE, UDP-galactose 4-epimerase; UGPUT, UTP-glucose-1-phosphate uridylyltransferase; PMM, phosphomannomutase-like protein; GalO, L-galactonolactone oxidase; PMI, phosphomannose isomerase; GNPI, glucosamine-6-phosphate isomerase; NagA, N-acetylglucosamine-6-phosphate deacetylase-like protein; GlyK, glycerate kinase; GarR, 2-hydroxy-3-oxopropionate reductase; DHAK, dihydroxyacetone kinase; DHAS, dihydroxyacetone synthase. **Pentose-phosphate pathway**: HPE, D-hexose-6-phosphate-1-epimerase; G6PDH, glucose-6-phosphate dehydrogenase; LAC, lactonase; 6PGD, 6-phosphogluconate dehydrogenase; RPI, ribulose-5-phosphate isomerase; R5PE, ribulose-5-phosphate epimerase; TKT, transketolase; SBTA, sedoheptulose-1,7-phosphate transaldolase; TAL, transaldolase; SBPase, sedoheptulose-1,7-bisphosphatase; RK, ribulokinase; RBK, ribokinase. **Gluconeogenesis**: FBPase, fructose-1,6-bisphosphatase. **Ether-lipid synthesis:** FAR, fatty-acyl-CoA reductase; ADHAPS, alkyl-DHAP synthase; DHAPAT, DHAP acyltransferase; AGPO, 1-alkyl G3P(NADP^+^)-oxidoreductase. **β-oxidation**: FACS, fatty-acyl CoA synthetase; ACAD, acyl-CoA dehydrogenase; ECH, enoyl-CoA hydratase; HADH, 3-hydroxyacyl-CoA dehydrogenase; 3-KAT, 3-ketoacyl-CoA thiolase; TECI, 3,2-trans-enoyl-CoA isomerase; ECI, enoyl-CoA isomerase; FAE, fatty acid elongase. **Sterol and isoprenoid synthesis**: HMCS, 3-hydroxy-3-methylglutaryl CoA synthase; HMCR, 3-hydroxy-3-methylglutaryl CoA reductase; MVK, mevalonate kinase; IDI, isopentenyl-diphosphate delta-isomerase; SQS, squalene synthase; SM, squalene monooxygenase; LDM, lanosterol 14-alpha-demethylase; SD, NAD(P)-dependent steroid dehydrogenase protein; SMT, sterol 24-C-methyltransferase; SI, C-8 sterol isomerase; 24-SR, sterol C-24 reductase; 3O5-SDH, 3-oxo-5-alpha-steroid 4-dehydrogenase. **Purine salvage**: NPase, nucleoside phosphorylase; APRT, adenine phosphoribosyltransferase; PRPPS, phosphoribosyl pyrophosphate synthetase; AMPD, AMP deaminase; IMPDH, inosine-5'-monophosphate dehydrogenase; GMPR, guanosine monophosphate reductase; HGPRT, hypoxanthine-guanine phosphoribosyltransferase; PPM, phosphopentomutase. **Pyrimidine synthesis**: OPRTase, orotate phosphoribosyltransferase; PRPPS, phosphoribosylpyrophosphate synthetase; OPDC, orotidine-5-phosphate decarboxylase. **Amino acid metabolism**: HAL, histidine ammonia-lyase; IPP, imidazolonepropionase; ProDH, proline dehydrogenase; P5CR, pyrroline-5-carboxylate reductase; T3DH, L-threonine-3-dehydrogenase; TDase, threonine dehydratase-like, OVDH, 2-oxoisovalerate dehydrogenase alpha subunit. **Defense against oxidative stress**: Argase, arginase; IDH, isocitrate dehydrogenase; GP, glutathione peroxidase-like protein; TR, trypanothione reductase; SOD, iron superoxide dismutase; AOD, acetylornithine deacetylase-like; Cyt B5 red, Cytochrome-B5 reductase; THR, thiol-dependent reductase 1. **Other enzymes:** AKR, aldo-keto reductase; NA, nicotinamidase. **Membrane (associated) proteins**: PEX, peroxin; GIM5A, glycosomal integral membrane protein 5. **Pore-forming protein**: PEX11. **Solute transporters:** MCF, mitochondrial carrier family protein; GAT, ABC transporter. **Membrane-associated enzyme**: CAT, carnitine/choline O-acyltransferase. For a detailed assessment of the results and a presentation of glycosomal metabolic pathways (see Acosta et al., 2019).

The proteome of *T. cruzi* glycosomes has recently been determined for the organelles isolated from cultured epimastigotes (Acosta et al., 2019). Many enzymes previously identified in glycosomes of *T. brucei* (Vertommen et al., 2008; Güther et al., 2014) and *Leishmania* spp. (Jardim et al., 2018) were also detected in the *T. cruzi* organelles: enzymes involved in glycolysis and gluconeogenesis with their auxiliary branches from phosphoenolpyruvate (PEP) comprising pyruvate phosphate dikinase (PPDK) and enzymes of the succinate production/utilization pathway, the pentose-phosphate pathway (PPP), biosynthesis of sugar-nucleotides, purines, and pyrimidines, sterols and ether-lipids and β-oxidation of fatty acids as well as enzymes involved in detoxification of oxygen radicals (Figure 2). Interestingly, also detected were enzymes for two possible novel routes for the reoxidation of the glycolytically produced NADH. The first route might involve a D-isomer specific 2-hydroxyacid dehydrogenase (HADH); this enzyme might catalyze the reduction of the pyruvate to lactate, whereas the enzymes for the second route are a putative aldehyde dehydrogenase (ALDH) that could reduce and decarboxylate pyruvate to acetaldehyde and an oxidoreductase (alcohol dehydrogenase, ADH) for the NADH-dependent reduction of acetaldehyde to ethanol (see also Figure 1 in Acosta et al., 2019). However, functional studies to prove these routes remain to be performed. Further identified, and characterized by our group were several enzymes previously reported to be present in *T. cruzi* but absent from *T. brucei*, such as a glucokinase (GlcK), galactokinase (GALK) and a unique form of phosphoglycerate kinase (PAS-PGK) with at its N-terminus a PAS domain, known to possess signaling and regulatory functions (Cáceres et al., 2007; Rojas-Pirela et al., 2018; Acosta et al., 2019).

Sterol synthesis is an essential metabolic process for *T. cruzi* and *Leishmania* spp. These trypanosomatids produce a special class of these lipids, including ergosterol and other C-24 methylated sterols, which are required for growth and viability of the parasites, but are absent from mammalian host cells (Soares and de Souza, 1991; Urbina, 1997). Previously, it has been shown that various enzymes of sterol synthesis in *T. brucei* and *L. major* are present in multiple intracellular compartments, including glycosomes (Carrero-Lérida et al., 2009). Various enzymes of the pathway were also detected in our proteomic analysis of *T. cruzi* glycosomes (Figure 2) (Acosta et al., 2019). Because of the unique aspects of the process in these parasites, several of its enzymes are being considered as a drug target (reviewed in de Souza and Fernandes-Rodrigues, 2009; Buckner and Urbina, 2012).

## Integration of Glycosomes in Overall *T. cruzi* Metabolism

Although glycosomes contain an extensive metabolic network, for most pathways only parts are present in the organelles (Figure 2). For glycolysis and gluconeogenesis the majority of the enzymes are compartmentalized, whereas for other pathways, such as the pentose-phosphate metabolism, purine salvage and biosynthesis of pyrimidines and ether lipids only some enzymes were detected in the organelles (Acosta et al., 2019). Moreover, enzymes showed often a dual distribution over glycosomes and cytosol, either due to partial compartmentalization (e.g., Concepción et al., 1999) or represented by distinct isoenzymes (e.g., Barros-Álvarez et al., 2014). This spatial organization of pathways implies that glycosomal metabolism is firmly embedded in the trypanosomatid's overall metabolism and that many metabolites that serve as substrates or products of glycosomal metabolism have to cross the membrane to connect them to the cytosolic parts of the pathways or for their further metabolism in other pathways located in different cellular compartments such as cytosol or mitochondrion. A well-studied example is the last part of the glycolytic pathway where either 1,3-bisphosphoglycerate or 3-phosphoglycerate exits glycosomes to the cytosol where it is converted via PEP to pyruvate by pyruvate kinase (PYK) that is then in part metabolized by pyruvate dehydrogenase and the tricarboxylic acid cycle in the mitochondrion. Alternatively, PEP enters the glycosomes for the production of succinate or pyruvate by the auxiliary branches of the glycolytic pathway mentioned above (Acosta et al., 2004). In addition, the succinate producing branch has a cytosolic shunt involving fumarate hydratase. The distribution of fluxes through these different pathways and the concomitant translocation of metabolites across the glycosomal membrane seem to be controlled by the requirement for maintaining an intraglycosomal balance between both ATP production and consumption and NAD^+^/NADH reduction and oxidation (Acosta et al., 2004).

This integration of glycosomal pathways in the trypanosome's overall metabolism has implications for the mechanisms by which metabolites cross the glycosomal membrane and may provide clues about the function of the organelles and the selective force that led to its evolution. These aspects will be discussed in the next sections.

## Solute Translocation Across the Glycosomal Membrane

Studies of metabolite translocation across the glycosomal membrane have been predominantly performed for *T. brucei*. These studies revealed a similar situation as for peroxisomes of different organisms: the existence of two groups of transporters, half-size ABC transporters and proteins of the Mitochondrial Carrier Family (MCF), as well as pore-forming proteins (reviewed in Gualdrón-López et al., 2013). Based on the situation in peroxisomes and some preliminary experiments with *T. brucei* glycosomes (Visser et al., 2007; Igoillo-Esteve et al., 2011; Antonenkov and Hiltunen, 2012; Gualdrón-López et al., 2012b), it was proposed that the ABC and MCF transporters are involved in the import of large molecules like fatty-acids and cofactors (ATP, NAD^+^, etc.) into glycosomes, while smaller molecules, with an estimated Mr of approximately 400 Da, such as glycolytic intermediates and inorganic ions pass through pores (Gualdrón-López et al., 2013). It has recently been shown in yeast that peroxin PEX11, previously identified as a factor involved in division and proliferation of peroxisomes from mammals, yeasts and plants, acts also as a protein in the formation of pores conducting solutes with Mr below 300–400 Da (Mindthoff et al., 2016). Trypanosomatid glycosomal membranes do contain PEX11 homologs. An additional pore-forming protein, Pxmp2, has been detected in mouse peroxisomes and functionally characterized. Electrophysiological experiments suggested that additional pore-forming proteins are also likely present in glycosomes but remain to be identified (Gualdrón-López et al., 2012b).

Proteomic analysis has revealed in *T. brucei* glycosomes other hypothetical membrane proteins which may be involved in translocation of other, notably large solutes, for example sugar-nucleotides, however this remains to be determined (Güther et al., 2014). The proteome of *T. cruzi* glycosomes showed a very similar repertoire of membrane-associated (candidate) transporters as in *T. brucei* (Acosta et al., 2019). Intriguingly, in glycosomes of both *T. brucei* and *T. cruzi* a putative carnitine O-acyltransferase (CAT) was found (Güther et al., 2014; Acosta et al., 2019). However, no acyl-carnitine translocase was detected that would suggest import of acyl-carnitines as alternative to that of acyl-CoAs via the ABC transporter GAT1 (Igoillo-Esteve et al., 2011) for providing acyl-CoAs for β-oxidation and ether-lipid biosynthesis. This situation seems reminiscent to mammalian and yeast peroxisomes for which it has been suggested that its CAT serves to produce acyl-carnitines from acyl-CoA derivatives that have been shortened by β-oxidation within the organelles. Carnitine esters are smaller than the corresponding CoA derivatives and may exit through the channels to the cytosol where they are converted back to free carnitine and acetyl(acyl)-CoAs by cytosolic and mitochondrial CATs (Antonenkov and Hiltunen, 2012). For a more detailed discussion about solute translocation across glycosomal membranes and transporters, see references (Gualdrón-López et al., 2013; Acosta et al., 2019).

The energization of metabolite transport through peroxisomal and glycosomal membranes has been a matter of debate for several years. It was clearly established that fatty-acid transport is mediated by ABC transporters driven by ATP hydrolysis at the cytosolic face of the organellar membrane (Wanders and Tager, 1998; Visser et al., 2007; Igoillo-Esteve et al., 2011). In contrast, data about the possible involvement of H^+^ or ion gradients to drive translocation of other solutes were contradictory (reviewed in Wanders and Tager, 1998; Antonenkov and Hiltunen, 2006). Several previous studies, using either radioactively labeled probes, often weak acids or bases, or fluorescent compounds containing a peroxisomal-targeting sequence (PTS), or by ^31^P-NMR, claimed the existence of a pH gradient across the membrane of these organelles. However, in some cases an intraorganellar matrix with alkaline pH compared to the cytosol was reported, while in other cases it was considered acidic. Furthermore, H^+^ or ion pumps or transporters for small solutes have so far never unambiguously been identified in any peroxisomal membrane (Antonenkov and Hiltunen, 2006, 2012; Visser et al., 2007). Recently, a fluorescein-tagged peptide containing a PTS has also been used in studies with procyclic *T. brucei* (Lin et al., 2013, 2017). The authors reported a slightly acidic intraglycosomal pH that was regulated independently from the cytosolic pH in cells subjected to variable nutrient availability. Based on inhibition studies, V-ATPases and Na^+^/H^+^ exchangers were invoked. However, no specific H^+^ or ion pumps have been identified thus far in glycosomes either, except one report of a V-ATPase in the proteome of bloodstream forms but not procyclic *T. brucei* (Colasante et al., 2006). Careful interpretation of proteome data about peroxisomes/glycosomes is required because it should be realized that these organelles, despite their high buoyant density, are notoriously difficult to isolate in intact form and free of contaminants from other organelles, because of their apparent high fragility (Antonenkov and Hiltunen, 2006) and, as has become apparent in recent years, their physical interactions with other organelles such as ER and mitochondria (Fransen et al., 2017; Kim, 2017; Shai et al., 2018). To obtain a high-confidence proteome of these organelles requires specific methods such as their enrichment by epitope tagging as done by Güther et al. (2014). Moreover, as has been argued (e.g., Antonenkov and Hiltunen, 2006, 2012), it is not clear how the presence of non-selective (with regard to anions vs. cations) transmembrane channels allowing permeation of solutes and ions with Mr < ~400 Da could be compatible with the active creation of pH gradients and membrane potentials across peroxisomal/glycosomal membranes. Additionally, the notion of a specific intraglycosomal pH is not obvious. Most peroxisomes are small spherical organelles; for bloodstream-form *T. brucei* an average diameter of 0.27 μm has been reported (Opperdoes et al., 1984; Tetley and Vickerman, 1991), with those in other trypanosomatids having comparable dimensions. This corresponds with a volume of 0.0108 μm^3^, implying that, at pH 7, on average, a glycosome would contain only 0.65 free H^+^ (i.e., using the definition of pH and Avogadro's number: 10^−7^ × 6.022 × 10^23^ × 0.0108 × 10^−15^ free H^+^/glycosome). The addition of a single free H^+^ would result in an intraglycosomal pH drop of ~0.4. It is important to realize that such calculations provide averaged values: averaged in time and space, over the total number of glycosomes in a trypanosome. Establishment of a pH is a dynamic process, involving metabolic reactions and the continuous protonation/deprotonation of many groups (of proteins, metabolites, phospholipids, etc.). One may thus wonder if the apparent response of the probes is affected by specific properties of the organelles, such as the high density of proteins with relatively high pI values—both considerably higher than in cytosol, the relatively high membrane/matrix ratio and/or the presence of inorganic polyphosphate (Negreiros et al., 2018). Because of the existence of non-selective, relatively large pores in the membrane, it seems realistic to consider that the cytosol and the matrix of the glycosomes form a continuum with regard to H^+^ and inorganic ions. Nonetheless, the higher density of proteins in the glycosomal matrix compared to the cytosol, the relatively high pI values of glycosomal enzymes and the presence of pores may be responsible for the creation of a Donnan equilibrium of H^+^, inorganic ions and small solutes (metabolites) across the membrane, i.e., slightly different concentrations on both sides, responsible for a (small) membrane potential and/or pH gradient. Furthermore, as argued by Antonenkov and Hiltunen (2006), the inconsistency in results obtained in different experiments for the intraperoxisomal pH may indicate it is a dynamic parameter depending on the physiological conditions in the cell. This is in line with the notion that some pH gradient across the peroxisomal membrane may be the created—transiently or constantly—by the Donnan equilibrium. Membrane-potential or pH-sensitive probes may thus respond to such differences (Gualdrón-López et al., 2012b; discussed in Antonenkov and Hiltunen, 2012).

## Biogenesis of Glycosomes

Glycosome biogenesis has been studied in detail in *T. brucei* and to a lesser extent in *Leishmania* spp. (reviewed in Galland and Michels, 2010; Gualdrón-López et al., 2013; Haanstra et al., 2016; Bauer and Morris, 2017). However, few studies have been devoted to this process in *T. cruzi*. The biogenesis occurs in a similar way as that of peroxisomes in other eukaryotes, involving mostly homologous proteins called peroxins or PEX proteins. Peroxins are involved in different stages of peroxisome formation, such as the posttranslational insertion of membrane proteins and import of matrix proteins. The organelles can be formed by two mechanisms: (i) budding from special parts of the endoplasmic reticulum (ER) after insertion of some (or all) peroxisomal membrane proteins (PMPs), followed by fusion with pre-existing peroxisomes and maturation involving matrix protein import, or (ii) PMPs and matrix proteins are directly sorted to existing peroxisomes. Lipids for growing and proliferating peroxisomes are provided by the vesicular transport from the ER, but may also be delivered via not yet well-studied non-vesicular routes involving contact sites between the organelles and the ER (Jansen and Van der Klei, 2019). The import of peroxisomal and glycosomal matrix proteins can occur by translocation of fully folded proteins and even multimeric complexes (Yang et al., 2018). This process starts in the cytosol by recognition of a peroxisomal-targeting signal (PTS), in most cases either a sequence motif at the C-terminus (PTS1) or one near the N-terminus (PTS2), in newly synthesized proteins by either of two cytosolic receptors of the peroxin family. The receptor-ligand complexes dock subsequently at the peroxisomal membrane where a series of interactions between different peroxins take place, culminating in the formation of a large transient pore through which the receptor-matrix protein complexes are imported into the organelles. The receptors are cycled back to the cytosol by an ubiquitin- and ATP-dependent mechanism to perform more rounds of import (Galland and Michels, 2010; Meinecke et al., 2010; Gualdrón-López et al., 2013).

A considerable number of *T. brucei* homologs of mammalian and yeast peroxins involved in PMP insertion and matrix protein import have been identified and characterized, and shown to be involved in glycosome biogenesis (Kalel et al., 2015, 2019; Banerjee et al., 2019; and reviewed in Galland and Michels, 2010; Gualdrón-López et al., 2013). Orthologous genes have been detected in the genome of *T. cruzi* and several of the proteins in its glycosomal proteome (Acosta et al., 2019).

## Polyphosphates in Glycosomes

Docampo and coworkers have shown that trypanosomes synthesize and store inorganic polyphosphate (polyP) in their acidocalcisomes. Recently, they also reported the presence of important amounts of long-chain polyP in the nucleolus and glycosomes of *T. brucei* and *T. cruzi*, where it interacts with proteins. In glycosomes it displays affinity to several enzymes of carbon metabolism (Negreiros et al., 2018). The reason for the presence of polyP in glycosomes remains to be established. Maybe it acts as a negatively charged scaffold in the creation of an assembly of the intraglycosomal enzymes which have generally a high pI and are present at high density (Misset et al., 1986). This notion is supported by the observation that glycosomal proteins remain largely packed in a complex when the membrane of purified glycosomes is dissolved by Triton-X-100 or permeabilized by digitonin; the complex is only disrupted at elevated ionic strength (Misset and Opperdoes, 1984; Misset et al., 1986). Alternatively or additionally, polyP may play a regulatory role in the enzyme activities, like PPi that has been shown to inhibit the activities of several glycosomal enzymes, such as *T. cruzi, L. mexicana* and one of the *T. brucei* hexokinases (HKs) (Cáceres et al., 2003; Pabón et al., 2007; Chambers et al., 2008) and *T. cruzi* PEP carboxykinase (Acosta et al., 2004), but this has not yet been tested for polyP. In addition, PPi plays a role as substrate or product in several reactions within glycosomes (Acosta et al., 2019). Interestingly, when a yeast exopolyphosphatase was expressed in *T. brucei* glycosomes, the intraglycosomal polyP levels decreased, while the glycolytic flux was also affected and the parasites became more susceptible to oxidative stress (Negreiros et al., 2018). Furthermore, the *T. brucei* genome contains genes encoding five proteins belonging to the Nudix superfamily, comprising enzymes that hydrolyse a wide range of organic pyrophosphates. Two of them, TbNH2 and TbNH4 possess polyP exopolyphosphatase and endopolyphosphatase activities, respectively. While TbNH4 localizes to the cytosol and nucleus, TbNH2 was detected in the glycosomes (Colasante et al., 2006; Güther et al., 2014; Cordeiro et al., 2019). TbNH2 has the tripeptide –SSI at its C-terminus, a possible PTS1 (Colasante et al., 2006), suggesting that the protein plays a role in polyP homeostasis in the organelles. However, no Nudix hydrolase was found in the glycosomal proteome of *T. cruzi* epimastigotes (Acosta et al., 2019). The *T. cruzi* genome encodes a TbNH2 ortholog (60% identical, 80% similar), but with a somewhat different C-terminal sequence (–SAL or –DSI, dependent on the strain) that may not be able to sort the protein to the organelles. Therefore, further research is required to establish if Nudix proteins are involved in glycosomal polyP hydrolysis. Whether polyP serves as glycosomal storage of PPi that is known to regulate the activity of several glycosomal enzymes seems questionable if PPi may easily pass through the pores in the membrane.

## Glycosomal Reprogramming During Differentiation of Trypanosomes

*T. cruzi*, like other dixenous trypanosomatid parasites, undergoes an elaborate life cycle involving extracellular replicative epimastigotes in the triatomine digestive tube, non-replicative extracellular metacyclic and bloodstream trypomastigotes in the insect and mammalian host, respectively, and replicative amastigotes intracellularly in the cytosol of the mammalian cells. The different developmental stages differ importantly in morphology, but also in metabolism to adapt to the large nutritional conditions encountered in the different niches (Maugeri et al., 2011; Barisón et al., 2017; Avila et al., 2018; Marchese et al., 2018; Mattos et al., 2019). Since glycosomes harbor many enzymes of intermediary metabolism as well as enzymes of other metabolic processes which are differentially expressed, glycosomal metabolism has to undergo reprogramming.

Indeed, levels and activities of glycosomal enzymes differ importantly between bloodstream-form and procyclic *T. brucei* (Hart et al., 1984). As for *T. cruzi*, a comparison of the proteome of glycosomes from epimastigotes harvested from the exponential and stationary growth phase, which, respectively, rely primarily on glucose and amino acids as carbon and energy source (Barros-Alvarez et al., 2014; Barisón et al., 2017), showed only small qualitative differences in the repertoire of both intraglycosomal enzymes and proteins in the glycosomal membrane (Acosta et al., 2019). It remains to be determined to what extent quantitative and/or qualitative differences occur in the glycosomal proteome during *in vivo* differentiation between the different life-cycle stages. Another recent study, in which *T. cruzi* trypomastigotes were analyzed before and after *in vitro* interaction with extracellular matrix (ECM), provides further information to the question to what extent quantitative and/or qualitative differences occur in the glycosomal proteome during *in vivo* differentiation between life-cycle stages (Mattos et al., 2019). Interactions with ECM components are essential prior to the invasion of mammalian host cells by *T. cruzi*. The authors reported important changes in the cellular phosphoproteome of the trypomastigotes, notably concerning proteins of carbon metabolism. A decrease of phosphorylation was observed for the glycosomal enzymes hexokinase (HK), phosphofructokinase (PFK) and PPDK, the cytosolic PYK, 6-phosphofructo-2-kinase/fructose-2-bisphosphatase (PFK2/FBPase2) and alanine transferase (ALT), and PGK that is present in both compartments, whereas phosphorylation of glycosomal fumarate reductase (FRD) was increased. These changes correlated with a decrease of activities of HK and PYK, as well as changes in the concentration of various intermediates of carbon metabolism. Together, the data indicate that phosphorylation of these enzymes, both glycosomal and cytosolic, plays an important role to control their activity and that the interaction of trypomastigotes with ECM components serves as trigger for the cells to prepare for intracellular life as amastigotes, by decreasing the glycolytic activity through dephosporylation of the enzymes. Previously, differential phosphorylation of proteins, including glycosomal enzymes, has also been demonstrated when bloodstream-form and procyclic *T. brucei* were compared (Urbaniak et al., 2013).

The mechanism by which the life-cycle stage dependent glycosome reprogramming occurs has not yet been studied in *T. cruzi*, but has been addressed in *T. brucei* and *Leishmania* spp. (Hart et al., 1984; Mottram and Coombs, 1985; Colasante et al., 2006; Herman et al., 2008; Vertommen et al., 2008; Brennand et al., 2011; Cull et al., 2014). Data indicate that this reprogramming involves mainly degradation of organelles containing the redundant enzyme repertoire by pexophagy—i.e., the autophagy process characteristic for peroxisome degradation—while new organelles are formed with an enzyme content that is appropriate for the conditions to be encountered by the new developmental form. Glycosomal proteins are encoded by genes in the nucleus and regulation of the glycosomal enzyme content is thought to occur via mechanisms controlling both expression of genes in general and that of genes for metabolic enzymes in particular in trypanosomes, i.e., mainly post-transcriptionally (Kafková et al., 2018; Clayton, 2019). Furthermore, it is feasible that the relative abundance of different proteins in the organelles is also dependent on differential rates of their import, since the sequence motif variants of PTS exhibit different import efficiencies (Sommer et al., 1992).

It has been proposed that compartmentalization of glycolysis and other core metabolic processes in glycosomes has evolved as a mechanism by which kinetoplastid organisms can adapt, by the turnover of the organelles through pexophagy and synthesis of new ones, their metabolism efficiently to large and sudden environmental changes as they encounter during their life cycle, possibly facilitating the emergence of a parasitic life style in many of these organisms (Herman et al., 2008; Brennand et al., 2011; Gualdrón-López et al., 2012a; Gabaldón et al., 2016).

Furthermore, in *T. brucei*, glycosomes have been shown to play a role in the parasite's differentiation. The organelles contain the serine/threonine phosphatase PIP39 with a PTS1 (–SRL) at its C-terminus. This protein is part of a protein phosphatase cascade that regulates the differentiation of the non-replicating short-stumpy bloodstream forms to the proliferating procyclic forms in the tsetse fly (Szöör et al., 2010). In the short-stumpy forms in the bloodstream, PIP39 is kept inactivated and dephosphorylated in the cytosol, by interaction with tyrosine phosphatase PTP1 at a periflagellar pocket location, closely associated with a flagellar pocket ER contact site, coincident with the location of a regulator of stumpy-form transcripts, REG9.1 (Szöör et al., 2019). When the parasites are taken up in the fly, the lowered temperature at ~20°C triggers an elevated expression of PAD proteins—members of the Major Facilitator Superfamily, most related to carboxylate transporters in other organisms—in the trypanosome's plasma membrane, allowing the uptake of citrate/cis-aconitate. This leads to disruption of the PTP1-PIP39 complex, PIP39 becoming phosphorylated and active, and relocated to glycosomes, whereas PTP1 becomes dispersed to a non-glycosomal, possibly cytosolic location. Szöör et al. (2019) proposed that the location of the “stumpy regulatory nexus” (STuRN) containing the PTP1-PIP39 complex and REG9.1 is similar to that of the pre-peroxisomal vesicles budding from the ER. After disruption of the complex, PIP39 is sequestered into glycosomes newly formed at this site. STuRN will so coordinate life-cycle differentiation with reprogramming of the glycosomal metabolic machinery.

*T. cruzi* contains a TbPIP39 homolog (59% identical, 77% similar, and also containing the PTS1 –SRL) that was detected in the proteome of epimastigote glycosomes (Acosta et al., 2019), as well as a TbPTP1 homolog (61% identical, 75% similar) (Szöör et al., 2006). It will be interesting to determine if PIP39 and PTP1 play a similar role, upon being triggered by a species-specific signal, in life-cycle differentiation of *T. cruzi* as they do in *T. brucei*.

## Conclusions

Glycosomes are authentic members of the peroxisome-organelle family, but have as distinguishable feature the sequestering of enzymes of glycolysis and other core processes of carbon metabolism. Although glycolysis is important, and in some trypanosomatid life-cycle stages essential for ATP production, there are several reasons why these organelles should not be considered as the cells “ATP factories” or as “energy-transducing organelles” comparable to mitochondria. First, ATP production and hydrolysis by all processes together—catabolic and anabolic ones—within the organelles seem to be kept in balance, net ATP production occurs outside the organelles. Contrary to mitochondria, there is no evidence for ATP delivery, in exchange for ADP, from the matrix to the cytosol. Also intraglycosomal NAD^+^ reduction and NADH oxidation are in balance; excess reducing power is transferred to the cytosol by a shuttle and from there to the mitochondrion. In contrast, mitochondrial shuttles serve to transfer electrons from cytosolic NADH to the organellar NAD^+^, mainly for subsequent oxidative phosphorylation. Translocation of adenine nucleotides and nicotine adenine nucleotides by transporters across the glycosomal membrane has not yet been demonstrated, but possibly occurs to provide cofactors, after their synthesis in the cytosol, to proliferating organelles, rather than to serve for balancing the stoichiometry of intraglycosomal metabolic processes. Second, there is no evidence that peroxisomal membranes are involved in energy transduction, like the inner-mitochondrial membrane derived from the cytoplasmic membrane of the ancestral endosymbiotic bacterium. Peroxisomal membranes originate mainly from the ER and incorporate proteins that form pores allowing the passage of molecules up to ~400 Da, but not the larger ATP, NAD(H) and other cofactors. It is therefore unlikely that they form a permeability barrier for H^+^ and inorganic ions. Import and efflux of metabolites occurs probably by diffusion through the pores and is dependent on their gradients, rather than transmembrane H^+^ or ion gradients, and on the control of the fluxes by the enzymes within and outside the organelles. Moreover, the notion of a separate matrix pH for small organelles as glycosomes is doubtful. Glycosomal function(s) should therefore rather be sought in other aspects such as assembly of enzymes in functional, efficient complexes and/or the coordinated turnover of major units of metabolic machinery by autophagy and biogenesis of the organelles during life-cycle differentiation (Herman et al., 2008; Brennand et al., 2011; Gualdrón-López et al., 2012a; Gabaldón et al., 2016).

An as yet unsolved question is how long-chain polyP is sequestered within glycosomes. There are no indications for its synthesis in these organelles. It is probably “piggyback” imported in association with the positively charged matrix proteins through the transient pores formed by peroxins which have the ability to transport proteins, even when folded, in multimeric form or with bound, large artificial ligands (Häusler et al., 1996; Meinecke et al., 2010; Yang et al., 2018).

Although the origin of peroxisomes and glycosomes is unrelated to that of mitochondria, it is important to realize that recent research has established contact sites, vesicular traffic, coordinated proliferation, and sharing of physiological functions between peroxisomes and mitochondria (Fransen et al., 2017; Kim, 2017; Shai et al., 2018). This aspect requires further study and has not yet been addressed for the organelles in trypanosomatids.

Finally, although glycosomes are evolutionarily related to peroxisomes, and share some functions, there are also considerable differences in enzyme content and aspects of their biogenesis. The large evolutionary distance between human and trypanosomes has resulted in large differences in the proteins involved. These differences, together with the essentiality of glycosomes and several of its metabolic processes make a variety of the trypanosomatid enzymes and peroxins promising drug targets. Indeed, selective, potent inhibitors of some glycosomal proteins have been developed that interfere with metabolic processes like sterol biosynthesis and glycosome biogenesis or function and kill the parasites (Buckner and Urbina, 2012; Barros-Alvarez et al., 2014; Dawidowski et al., 2017).

## Author Contributions

The experiments to create Figures 1A–D were performed by CG and MM, those for Figure 1E by MG-L, HA, and WQ made Figure 2. Each of the authors contributed to the design and preparation of the manuscript, and approved it for publication.

### Conflict of Interest

The authors declare that the research was conducted in the absence of any commercial or financial relationships that could be construed as a potential conflict of interest.
